# Chronic stress influences attentional and judgement bias and the activity of the HPA axis in sheep

**DOI:** 10.1371/journal.pone.0211363

**Published:** 2019-01-30

**Authors:** Else Verbeek, Ian Colditz, Dominique Blache, Caroline Lee

**Affiliations:** 1 CSIRO, Agriculture and Food, Locked Bag 1, Armidale NSW, Australia; 2 School of Agriculture and Environment and the University of Western Australia Institute of Agriculture, The University of Western Australia, Crawley WA, Australia; Radboud University Medical Centre, NETHERLANDS

## Abstract

**Introduction:**

Environmental challenges are part of everyday life for most domestic animals. However, very little is known about how animals cope emotionally and physiologically with cumulative challenges. This experiment aimed to determine the impact of long-term exposure to environmental challenges on the affective state and hypothalamic-pituitary-adrenal (HPA) axis responses to a subsequent additional acute shearing challenge.

**Methods:**

Sheep were exposed to either a long-term environmental challenge (rest disruption and individual housing) in order to induce chronic stress (chronic stress group) or control conditions (group housing in a field with low stress handling and daily feed rewards, control group). Judgement and attention bias were assessed as measures of the emotional state following several days of the challenge or control treatment (pre-shearing tests). In addition, the responsiveness of the HPA-axis was evaluated using a combined Corticotropin Releasing Hormone and Arginine Vasopressin (CRH/AVP) challenge. Finally, all animals were exposed to an acute shearing challenge, then judgement bias (post-shearing test), HPA-axis and internal body temperature responses were determined.

**Results:**

In the pre-shearing judgement bias test, the chronic stress group slightly increased optimism compared to the control treatment. In the attention bias test, the chronic stress group showed reduced vigilance behaviour towards a predator threat and a quicker approach to the food compared to the control treatment. The chronic stress group also had lower plasma ACTH concentrations in response to the CRH/AVP challenge compared to the control group, no differences in cortisol concentrations were found. In the post-shearing judgement bias test, differences in optimism were no longer evident between the chronic stress and control groups. Plasma ACTH concentrations and body temperatures showed a greater increase in response to shearing in the chronic stress group compared to the control group.

**Conclusion:**

Our results suggest that long-term exposure to challenges biased cognitive measures of the affective state towards an increased expectation of a reward and reduced attention towards a threat. The exaggerated ACTH responses in the chronic stress group may be indicative of HPA-axis dysregulation. Despite a period of challenge exposure in the chronic stress group, judgement bias responses to the shearing challenge were similar in the chronic stress and control groups; the reasons for this need further investigation. The altered affective state together with signs of HPA-axis dysregulation may indicate an increased risk of compromised welfare in animals exposed to long-term environmental challenges.

## Introduction

Environmental and psycho-social challenges are encountered on a regular basis throughout an animal’s life. Captive animals, such as farm, zoo and laboratory animals may experience several simultaneous challenges, such as barren housing conditions, painful husbandry procedures, rest deprivation and other stressful rearing and handling practices. However, very little quantitative evidence is available on how repeated or continuous exposure to challenges alters the emotional state of animals, which is an important aspect of their welfare. Furthermore, little knowledge exists on how long-term exposure to environmental challenges modulates emotional and physiological adaptation to additional challenges.

In response to a challenge, the hypothalamic-pituitary-adrenal (HPA) axis is activated allowing the individual to physiologically and behaviourally respond to the challenge. This is an adaptive response through which the individual attempts to cope with the challenge and regains energy homeostasis [1, 2]. Adjustments in internal body temperature also occur in response to challenges and may be a useful indicator of adaptation to the challenge [3]. However, if the challenge is prolonged or occurs repeatedly, it can eventually result in chronic changes in the behaviour and physiology of the animal; a biological state often referred to as chronic stress. A protracted redirection of biological resources away from biological activities that were occurring before the challenge is a hallmark of failure to adapt and characterizes stress [4, 5]. The impact of the challenge varies depending on the predictability, controllability, ambiguity and chronicity of the challenge [6, 7]. A chronic stress state is accompanied by a number of neuro-chemical changes such as hyper- or hypo-activity of the HPA-axis, down regulation of the HPA-axis and alterations to a number of receptor systems [6, 8]. The excessive wear and tear (allostatic load) on homeostatic pathways during chronic stress may eventually lead to dysregulation of the HPA-axis [2, 9]. Such maladaptive stress responses can reveal themselves in multiple ways, such as lack of adaptation to a challenge, a prolonged response to a challenge or an inadequately low response to a challenge [2]. Exposure to long-term challenges can eventually lead to mood changes [9] and it has been hypothesised that maladaptive stress responding may increase the risk of developing depressive or anxious emotional states [2, 6].

Long-term or repeated exposure to challenges may also have consequences for the ability to adapt to novel events or additional challenges, due to the already increased allostatic load of an individual under stress [10]. However, very little is known about the influence of long-term challenges on emotional and HPA-axis responses to subsequent challenges. Sheep exposed to 6 weeks of unpredictable and uncontrollable events were shown to have increased fearfulness [11]. Also in sheep, barren and unpredictable housing led to a stronger behavioural response (altered proportion of the asymmetric ear posture, inter-heartbeat interval, respiration rate) to both negative (social separation) and positive events (grooming) compared to sheep in a more positive state [12]. Sheep housed in a barren environment also showed a stronger cerebral response to grooming [13]. Furthermore, undernourished sheep showed reduced metabolic and stress responses to a cold challenge [14]. Chronic sleep deprivation also reduced the HPA-axis responses to an additional psychological stressor in rats, and led to similar neuro-chemical changes as observed in depression [15].

In animals, research on affective states is limited because there is no direct objective measure of the affective state. Human psychological studies have shown that there is a bidirectional link between cognition and affect; the affective state of an individual influences cognitive processing and alters how information from the environment is perceived and interpreted [16, 17]. Using this link between cognition and affect, a cognitive (judgement) bias test has been developed that provides an indicator of the affective state in animals [18]. A judgement bias is presumed to exist when the animal behaves as if it has an increased expectation of a punishment (pessimism) or an increased expectation of a reward (optimism) when encountering ambiguous information [19]. It has been shown that exposure to uncontrollable and unpredictable events led to a pessimistic judgement bias in rats and sheep [18, 20]. In contrast, environmental enrichment led to an optimistic judgement bias in starlings [21] and rats [22], while a food reward after morphine administration led to an optimistic judgement bias in sheep [23]. Therefore, the judgement bias methodology appears to measure at least some aspects of the affective state in animals.

Other cognitive measures of the affective state are relatively unexplored in mammals, such as heightened vigilance behaviour directed towards a threat, also called an attention bias [19]. An attention bias towards threatening information is likely to facilitate faster detection of a threat and hence faster appropriate behavioural and physiological responses to fight or escape the threat, and would hence provide a fitness advantage [24, 25], which may be particularly relevant for animals living in a challenging environment. In humans, it has been observed that anxious individuals bias their attention towards threats [26], and there is evidence that such biases also exist in animals. A study in rhesus macaques (*Maccaca mulatta*) showed that they reduced their attention towards aggressive faces when subjected to stressful procedures [27]. Starlings (*Sturnus vulgaris*) were more vigilant and less likely to feed when hearing an alarm call when their fitness was reduced [28]. Based on the starling study, we have developed a test to assess attention bias of sheep and shown that sheep treated with the anxiogenic drug m-CPP showed increased vigilance and attention towards a predator threat, while sheep treated with the anxiolytic diazepam showed the opposite responses [29]. This modulation of attention towards a predator threat suggests that attention bias can assess different levels of anxiety in sheep.

The main aim of this experiment was to determine the ability of sheep exposed to a long-term environmental challenge, and therefore in a chronic stress state, to cope with an additional acute shearing challenge. To answer this question we first determined the emotional state and HPA-axis responses of sheep subjected to individual housing and unpredictable and uncontrollable rest disruption, compared to sheep kept together as a group in an environment with minimal challenges. We hypothesise that chronically stressed sheep will express a more pessimistic judgement bias, an increased attention bias towards a predator threat and an increased HPA-axis response to a combined CRH/AVP challenge. Following this, we subjected sheep of both treatments to an acute shearing challenge. We hypothesised that immediately following the acute shearing challenge, the chronically stressed animals will show an even more pessimistic judgement bias, an increased HPA-axis response and increased stress-induced thermogenesis. The results of this study will contribute to a better understanding of repeated exposure to environmental challenges and affective states in farm animals as a welfare aspect of animal husbandry. Furthermore, this study contributes to a better understanding of the processes underlying changes in judgement and attention bias.

## Methods

### Animal ethics

This study was approved by the Chiswick Animal Ethics Committee (Animal Research Authority #12–14). All animals were closely monitored during and after the experiments and no long-term effects on animal health and welfare were observed.

### Animals and management

Sixty Merino ewes (11–12 months of age, 35.6 ± 0.5 kg at the start of the habituation period at day -83 in Table 1) that had never been shorn were used in this experiment. All ewes were born on the same experimental farm and reared as one group after weaning. At the start of the habituation period (day -83, Table 1), all ewes were grazed on pasture and sheep were slowly introduced to dietary supplementation over a period of two weeks. Supplementation consisted of sheep concentrate pellets (containing wheat, lucerne, pollard, bran, salt and ammonium chloride with 11.9 MJ/kg DM, 22% crude protein, 2.5% fat, 11% ash, 14% acid detergent fibre and 30% neutral detergent fibre) and oaten chaff (8.9 MJ/kg DM with 11% crude protein, 9% ash, 33% acid detergent fibre and 57% neutral detergent fibre). After the two weeks of habituation to the food animals were fed 800 g pellets and 200 g chaff per day. We aimed for an equivalent energy intake for animals in both treatments, even though housing conditions were not the same. Sheep were weighed once a week throughout the experiment.

**Table 1:** Experimental design and time line.

	Treatment Group		
Day	Chronic stress	Control	Comfort times for chronic stress group*
-83	Habituation to handlers and feed supplementation		
-69	46 sessions of judgement bias training Sheep run as one group in a paddock adjacent to training facility		
1	Commence lying disruption and individual housing	Continue management as a group of 15 ewes	10:00–16:00 h
2		Measure time spent lying (measured between 10-15h and 16-8h)	12:00–18:00 h
3			14:00–20:00 h
4	Measure time spent lying (measured during comfort time 10-15h and deprivation time 16-8h)		9:30–15:30 h
5			9:30–15:30 h
6	Judgement bias test		17:00–23:00 h
7	CRH/AVP challenge		12:00–18:00 h
8	Attention bias test		14:00–20:00 h
9	Shearing, then judgement bias test, Cortisol/ACTH assessment, body temperature monitoring		End of experiment

* Indicates the time of day when the chronic stress group was allowed to lie down comfortably (6 hours/day).

### Experimental design and treatments

Sheep were trained over a period of two months to undertake a judgement bias test (see below), animals remained on pasture during the training period. The 30 sheep that reached the learning criteria in the judgement bias test (see below) were pseudo-randomly split into two groups of 15 animals each, which were allocated to chronic stress and control treatments.

The chronic stress group was housed in individual pens (0.9 x 2 m) in an indoor sheep facility and restricted from lying comfortably for 18 h/day for a period of nine days. An uncomfortable lying surface comprised of a 0.9 x 2 x 0.14 m wooden grid was placed on the floor in each pen during the lying restricted times. Previous research has shown that seven days of lying restriction was sufficient to induce chronic stress response in dairy cows [30]. A vacant pen between each sheep prevented physical interactions but permitted visual and auditory contact between animals. Sheep were moved into an adjacent pen without the grids for a six hour interval each day at unpredictable times (often during daylight hours), thus allowing access to a metal mesh floor to lie on. Contact between handlers and animals was kept to a minimum and restricted to the most necessary husbandry procedures (e.g., feeding, weighing, etc). The chronic stress group also received an additional 200 g of oaten chaff (total 400 g oaten chaff) in order to ensure sufficient roughage intake and to compensate for the possibility of a small amount of grass intake in the control group (see below).

The control group was kept in a paddock grazed bare of feed (in order to prevent confounding effects of diet) adjacent to the judgement bias test facility. Control sheep also received two small palatable rewards (total 50 g of a lupin and barley mixture) per day; once after an experimental manipulation had been applied (e.g., a blood sample had been collected) and once at a different time while in the paddock. The animals were handled gently (i.e., use of calm voice, no sudden movements, quiet environment etc) and were allowed to interact positively with the handlers if the sheep chose to do so (e.g., voluntary grooming).

The measurement protocol is summarised in Table 1. Commencement of chronic stress and control treatments was called day 1. Activity sensors (IceQubes, IceRobotics, South Queensferry, UK) were fitted to the right front leg of the Control group (day 2 and 3) and the chronic stress group (day 4 and 5) to measure the time spent lying. As insufficient devices were available to measure lying behaviour of all animals simultaneously, a serial design was used so that we could ensure that data was collected during the same daytime hours (between 10:00 h and15:00 h comfort time and between 16:00 h and 8:00 h the next day for restricted time) for both treatments (the chronic stress group was lying restricted during this period on all other days to induce the chronic stress, see Table 1) on 2 different days to account for variation in diurnal patterns of activity with time of day and also between days. Judgement bias was measured on day 6 and a combined CRH/AVP challenge was conducted on day 7. On day 8, attention bias was assessed and after this sheep were fitted with intravaginal temperature loggers. On day 9, sheep were exposed to shearing as an acute stressor. Body temperatures, ACTH and cortisol concentrations were monitored before and after shearing, and immediately after shearing judgement bias was assessed. Animals continued to receive their chronic stress or control treatments until all testing was completed at the end of day 9.

### Judgement bias arena

**Fig 1:**
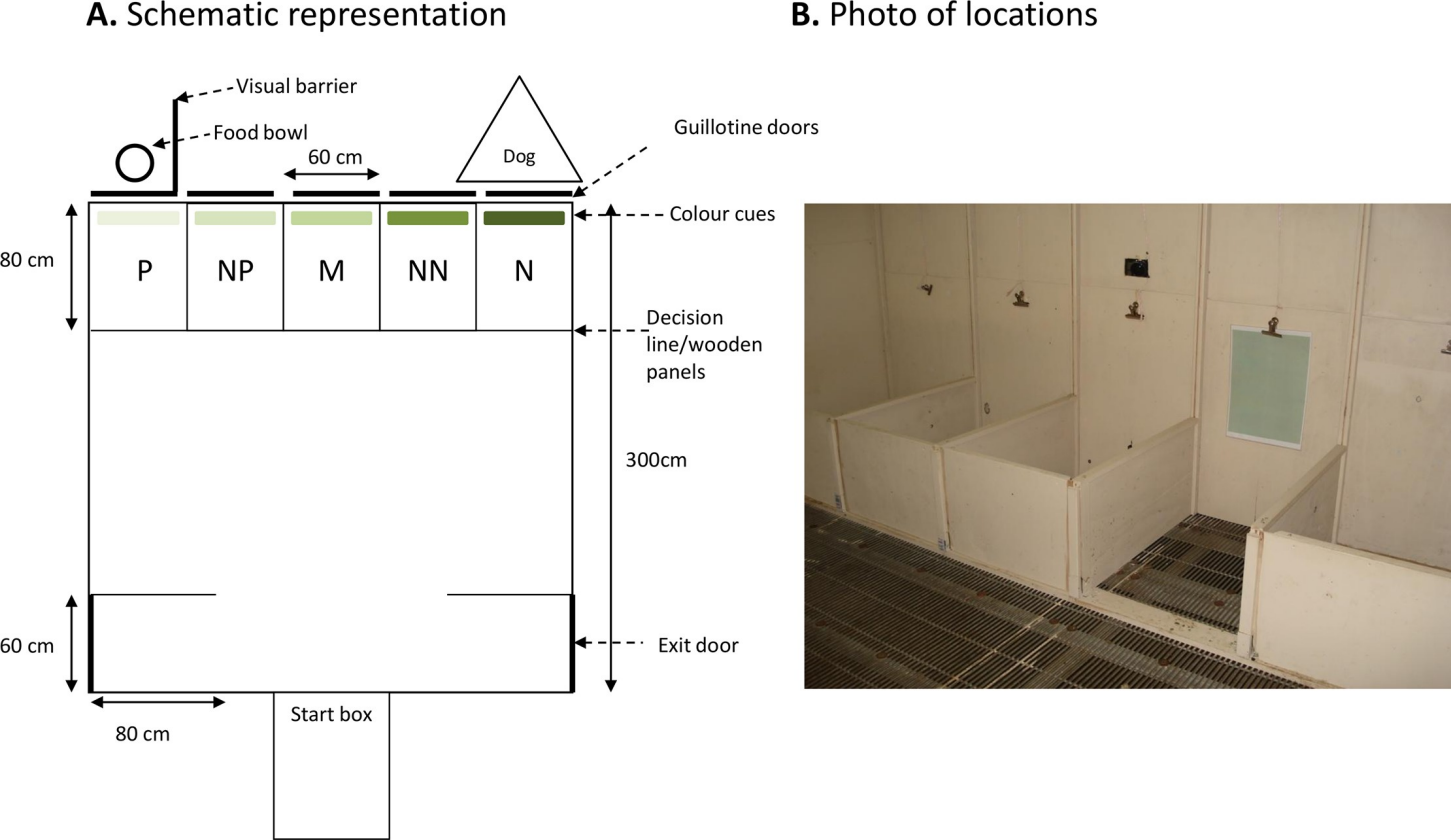
Judgement bias arena. The arena has five different cued locations (positive (P), near positive (NP), middle (M), near negative (NN) and negative (N) locations), shown as A. schematic representation and B. as photo of the actual experimental setting. Different colour cues (shades of green) could be attached to the sliding doors (total five different location and colour cue combinations). The figure is an example for a sheep trained with left positive combined with a 95% transparency colour cue. Only one cued location was accessible per arena entry (the photo shows an example of the NN cued location accessible). Part B reprinted from [33] with permission from Elsevier.

A spatial differentiation task that had previously been validated for testing judgement bias in sheep was adapted to use food as a positive reinforcer (see [31] for more details). Judgement bias was assessed in a 3 x 3 m arena surrounded by solid wooden walls (1.5 m high). The back wall of the arena was divided into five boxes, from now on called locations (Fig 1). The front of each box could be opened. Behind the two most outer locations was a vertical sliding door that could be lifted to reveal either a dog or a bucket of food (negative and positive reinforcers, respectively, see below). Identical dummy sliding doors were placed behind the three middle locations (ambiguous locations, see below), but these could not be physically opened. In order to facilitate discrimination between the different locations, colour cues printed on A3 size laminated paper could be attached to the sliding doors. The cues were all the same colour green, but differed in colour density (0, 25, 50, 75 and 95%) that was adjusted by changing the colour transparency in Microsoft PowerPoint. We chose different densities of the same colour green, because previous research has shown that sheep have a good ability to discriminate between different degrees of brightness [32]. Therefore, there were a total of five different location and colour cues used for judgement bias assessment: positive (P, closest to the food bucket), and then in order, near positive (NP), middle (M), near negative (NN) and negative (N, closest to the dog).

### Judgement bias training

Sheep received a total of 46 training days, spread over a period of 2 months and 9 days, using a method adapted from a previous study [31]. A detailed description of the training can be found in the S3 File. After a short habituation period, sheep were pseudo-randomly assigned to a Positive (P) location in either the outer left or outer right corner of the arena and were individually trained to approach a reward in a bucket (the concentrate pellets sheep were normally fed) at the P location while all other locations were inaccessible. Sheep always entered the arena five times within one training session, and they received one training session per day. A ‘positive response’ was considered correct when the sheep stepped over the decision line at the entrance of the location with both front feet (Fig 1) and put its head inside the bucket within 30 s of entering the test arena (go response). As soon as the animals had consumed the reward, they were let out of the arena. If the animal did not make an approach within the maximum available time (30 s), they were also let out of the arena (no-go response). Sheep had to pass a performance criterion of 15 consecutive correct positive responses to the P location in order to proceed to negative training. Cue cards were not displayed above locations at this stage of training.

‘Negative training’ involved placing the bucket at the N location in the opposite corner of the arena (if the P location was the outer left corner, then the N location was the outer right corner), although it contained no food. A dog was lying quietly behind the sliding door and was only revealed when the sheep stepped over the decision line with both front feet (Fig 1) by lifting the sliding door (incorrect negative ‘go response’). Sheep were let out of the arena after about 5 s of exposure to the dog, to prevent the sheep getting too stressed. The dog was visible from all areas of the arena. A correct negative ‘no-go response’ was made when the sheep did not approach the N location, and in this case the animal was let out of the arena after 30 s. The dog always remained in position, but with the sliding door closed, even when sheep were being trained at the P location.

After several days of positive and negative training, many sheep continued to approach the N location, despite exposure to the dog when they approached the empty bucket. Therefore, the bucket was removed from the test arena. Instead, the food reward at the P location was placed in a bowl behind the sliding door. Sheep had to step over the decision line within 30 s of entering the arena in order for the door to be lifted and the food to be accessible. To facilitate discrimination between the different locations, the colour cue cards were attached to the sliding doors at the different locations. Sheep were pseudo-randomly assigned to either a 0% (white) or 95% brightness green cue at the P location. For each sheep, the cue at the N location was the opposite of the P location.

Finally, sheep had to pass a criterion in which they had to approach the P location 14 out of 15 times (go response), and not approach the N location at least 8 out of 10 times (no-go response) within 30 s (minimum of five sessions on five different days).

Out of 60 sheep, 32 sheep passed all training criteria. Of these, two were randomly excluded and the remaining 30 sheep were used in the experiment. Based on previous experience [31, 34]; 15 animals per treatment is sufficient to observe statistically meaningful differences in this test and it was therefore considered unnecessary to subject additional animals to the experimental procedures.

### Judgement bias assessment

Judgement bias was assessed following six days of individual housing and rest disruption (pre-shearing test) and again immediately after shearing (post-shearing test). Animals were released into the arena five consecutive times, with the two trained cue locations and the three ambiguous cued locations each presented once. The two trained locations were reinforced by a food reward (P) or exposure to the dog (N) while the ambiguous locations were not reinforced. Half the animals within a treatment were randomly assigned to the following order of cued locations: P, NP, M, NN and N (P order) and the other half to N, NN, M, NP and P (N order), so that the sequences were balanced across treatments in order to minimize the effects of cue on subsequent choices. Our experimental design also ensured that animals from both treatments were evenly spread throughout the testing day. An animal was considered to have made a go-response if it approached the cued location within 30 s. The go or no-go responses were recorded for each location from a video screen in real time. All tests were continuously recorded by two cameras (SCC-B2315P, Samsung, Seoul, South Korea) and a 16 Channel Digital video recorder (PACOM, Port Melbourne, Victoria, Australia).

### CRH/AVP challenge

A baseline blood sample was taken at time 0 and immediately afterwards sheep were injected with 0.5 μg/kg body weight ovine CRH and 0.1 μg/kg arginine vasopressin (Sigma-Aldrich, St Louis, USA). Blood samples were collected by jugular venepuncture into EDTA vacutainers at 20, 40 and 60 min and centrifuged at 3000 rpm immediately after collection. Samples were stored at -80°C until analysis for plasma cortisol and ACTH concentrations.

### Attention bias assessment

Attention bias was measured as described in [29]. Briefly, an unfamiliar 4m x 4m arena surrounded by a 1.5m high fence covered in opaque shade cloth was used. The floor of the arena was divided into 12 square zones (1 m x 1 m) painted in white paint and a bowl with concentrate pellets was placed in the centre. On one side of the fence, an unfamiliar dog was sitting behind a small opening for the first 30 s of the test. After 30 s, the opening was closed, the dog was removed and the sheep was left in the arena for an additional 3 min. The following behaviours were recorded: number of zones crossed, number of vocalizations, latency to reach the food bowl, amount of food eaten, duration looking at the dog and duration looking at the food. Vigilance behaviour was defined as the head at shoulder height or higher and the total duration of vigilance behaviour was recorded as well as the frequency of transitions between vigilance and non-vigilance behaviour.

### Body temperatures

Temperature loggers (Thermochron temperature loggers, model DS1921H-F5, Thermochron, Baulkham Hills, Australia) attached to non-impregnated Eazi Breed CIDRs (intravaginal Controlled Internal Drug Release Devices, Zoetis, Rhodes, Australia) were inserted into the vagina after attention bias testing on Day 8. The loggers were programmed to log temperature every minute. Prior to the study, the accuracy of the loggers (logging temperature every minute) was assessed by placing them in a hot water bath at 30°C for 15 min and then at 40°C for an additional 30 min. Variation between loggers at both the minimum and maximum temperatures averaged 1°C ± 0.25 (mean ± Standard Deviation).

### Assessment of responses to shearing challenge

Sheep from both treatments were shorn by an experienced shearer at time intervals spread evenly throughout the testing day. Blood was collected by jugular venepuncture into EDTA vacutainers at time 0 immediately before shearing and 20 and 40 min later. Tubes were spun at 3000 rpm for 15 min, and plasma collected and stored at -80°C until analysis for plasma cortisol and ACTH concentrations. Immediately after shearing, judgement bias was assessed again as described above (post-shearing test).

### Blood sample analysis

Plasma cortisol concentrations were measured using a commercial radioimmunoassay (Orion Diagnostica, Espoo, Finland), previously validated for ovine plasma cortisol in the laboratory [35]. The three quality controls (QCs) contained 27.5, 65.1 and 121.7 nmol/L of cortisol with intra-assay coefficient of variation (CV) of 8.5%, 7.1%, and 11.0%, respectively.

Plasma ACTH concentrations were measured in duplicate 100 μl aliquots using a human ACTH immunoradiometric kit (Diasorin Australia Ltd, North Ryde, NSW) previously validated for ovine plasma ACTH [36]. Extra standards were made by diluting standards with the zero standard to extend the limit of detection of the assay was 3.1 pg/mL. Three control samples containing 35.8 pg/mL, 107.8 pg/mL and 309.1 pg/mL were included in the assays and were used to estimate the intra-assay coefficients of variation of 1.6%, 1.5% and 0.9% and the inter-assay coefficients of variation of 4.0%, 2.1% and 1.0%.

### Statistical analysis

Data are presented as (back-transformed) predicted means [95% confidence interval], unless stated otherwise. Assumptions of normal distribution and homoscedasticity were assessed by graphical analysis of the residuals, and data were log-transformed (cortisol responses to CRH/AVP and shearing challenges, ACTH responses to the shearing challenge, and the maximum body temperatures) or square root-transformed (latency to eat, food intake, the number of zones crossed and vocalizations) if necessary, after which assumptions were met. Data was analysed in R using package lme4 for generalized linear mixed effect models [37].

We used an information criterion based approach for model selection that measures the strength of evidence for each model within a given set of models [38]. The advantage of using this approach that does not use P-values to determine statistical significance, is that it reliably and objectively measures the strength of evidence for each model within a given set of models. The measures we used (see below) also provide evidence for the strength of each model so that an optimal model (or models) can be defined. This makes significance testing and presenting P values redundant. This approach has also been proposed as an appropriate way to analyse judgement bias data [39].

We defined a set of fixed effects models *a priori* and used the Akaike Information Criterion adjusted for small sample sizes (AICc) to select the most appropriate model based on Δ_*i*_ (AICc differences relative to the smallest AICc value in the set of models, a Δ_*i*_ of less than 4 suggests that models are very similar in terms of their optimality) and *w*_*i*_ (AICc weight), the probability of the model being the expected optimal model for the situation considered [40]. The “maximum” model in our set of models included all potential predictors and their interactions. We used the function “dredge” in package “MuMIn” to automatically calculate the AICc parameters for the different combinations of predictors and their interactions [41]. The “minimal” model consisted of the intercept only. In case of one particular model having a high model probability and the others have a probability close to zero, there is considerable evidence for the model. However, often several models have similar model probabilities (and a Δ_*i*_ of less than 4), which means that all these models could potentially explain the data.

The IceQubes recorded lying behaviour as the number of minutes per hour spent lying. For analysis, we divided the data into two time periods (one data point per individual and period): a comfort (non-lying deprived) period of 5 hours (from 10:00 h until 15:00 h to account for slight variation in comfort times) and a lying deprived period of 16 hours (from 16:00 h until 8:00 h the next morning). Lying behaviour was analysed by a generalized linear mixed effect model with time period, treatment and their interaction as fixed effects and ewe as a random effect in the maximum model. For the analysis of body weight, treatment, time and their interaction were included as fixed effects and sheep as a random effect in the maximum model. The maximum model for cortisol and ACTH responses to the CRH/AVP and shearing challenges included treatment, time and their interaction as fixed effects and sheep as a random effect. The Area Under the Curve (AUC) for the cortisol and ACTH responses included treatment as a fixed effect in the maximum model. Body temperatures were measured as the change from the baseline at 10 min prior to shearing, in order to account for the variation in temperature recordings between individual temperature loggers (see above). First, temperature data was analysed from 0 to 50 min (2 minute intervals) after shearing, with treatment, time and their interaction as fixed effects, and sheep as a random effect in the maximum model. In addition, the highest individual recorded temperatures (peak) was analysed with treatment as a fixed effect. The binomial go/no go data from the two judgement bias tasks were analysed by a logistic generalised linear mixed model with a logit link function, with location, treatment, and their interactions as fixed effect and sheep as a random effect in the maximum model.

## Results

### Effects of individual housing and rest disruption

#### Lying behaviour and body weight

For time spent lying down, there was strong evidence for a model that fitted a treatment x lying deprivation interaction (Δ_*i*_ = 0, *w*_*i*_ = 1), with the chronic stress group spending more time lying down (predicted mean [95% confidence interval]: 44.4 [40.3; 48.5] min/h) than the control group (15.6 [11.7;19.6] min/h) during comfort time, while the treatment difference during the lying disruption period was small (26.4 [22.3; 30.5] min/h for the chronic stress group and 30.3 [26.3; 34.2] min/h for the control group).

There was weak evidence for a slightly greater decrease in body weight in the chronic stress group than in the control group (best model included a time x treatment interaction, (Δ_*i*_ = 0, *w*_*i*_ = 0.37), followed by a model including the main effect of time only (Δ_*i*_ = 0.17, *w*_*i*_ = 0.34); one week prior to commencement of the treatments the chronic stress group weighed 35.7 [34.2; 37.2] kg and the control group 35.6 [34.1; 37.1] kg. One week after completion of the treatment the chronic stress group weighed 33.8 [32.3; 35.3] kg and the control group 34.4 [32.9; 35.9] kg.

#### CRH/AVP challenge

**Fig 2:**
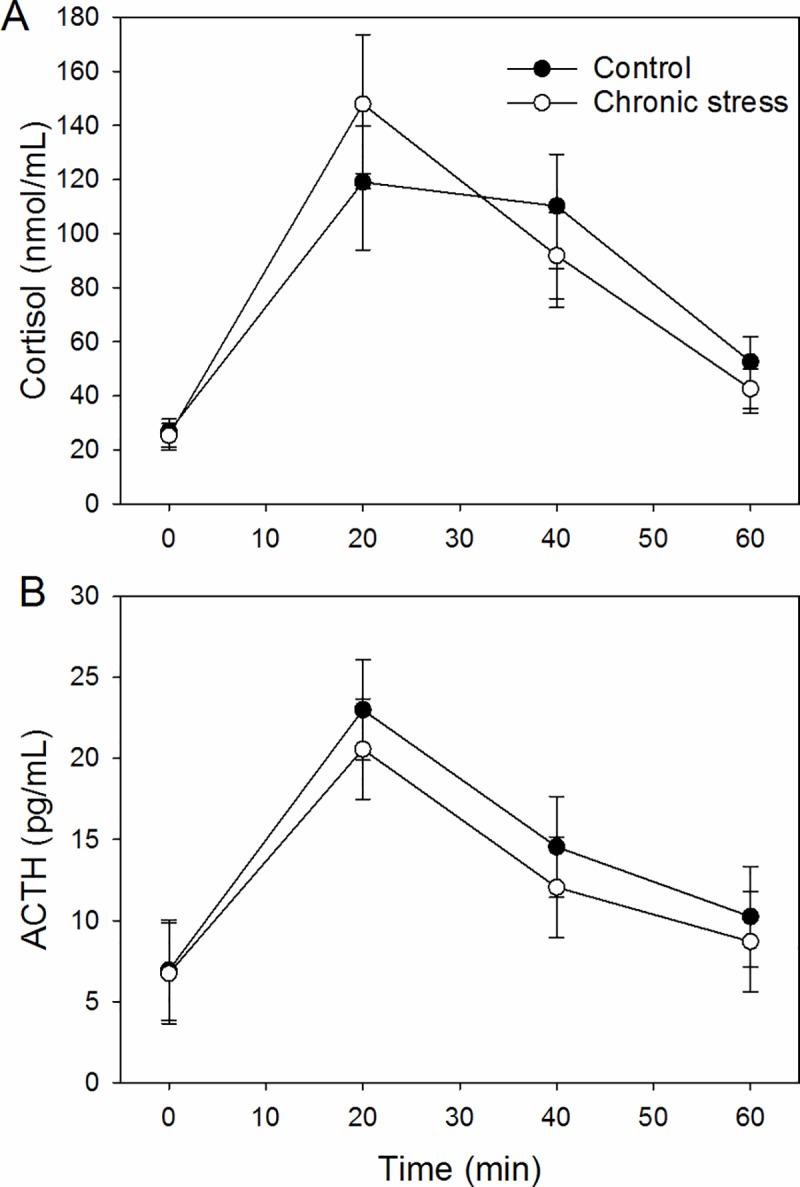
Effects of chronic stress treatment on predicted mean ± confidence interval plasma cortisol (A) and ACTH (B) concentrations during the CRH/AVP challenge.

The cortisol responses to the combined CRH/AVP challenge were best explained by the time course of the experiment (model fitting the main effect of time only: Δ_*i*_ = 0, *w*_*i*_ = 0.96) with no evidence for a treatment effect on the cortisol responses (model fitting main effects of time and treatment: Δ_*i*_ = 6.4, *w*_*i*_ = 0.04, Fig 2A). The optimal model for the Area Under the Curve (AUC) of the cortisol response was the intercept only model (Δ_*i*_ = 0, *w*_*i*_ = 0.77), and we only found weak evidence for an effect of treatment (chronic stress group 96.2 [84.6; 107.8] ng.ml^-1^/min and control group 93.5 [81.9; 105.1] ng.ml^-1^/min, Δ_*i*_ = 2.36, *w*_*i*_ = 0.24).

The chronic stress group’s ACTH response to the combined CRH/AVP challenge (Fig 2B) was lower than the control group (best model fitting a time x treatment interaction: Δ_*i*_ = 0, *w*_*i*_ = 0.83, the second best model fitted the main effects of time and treatment Δ_*i*_ = 3.83, *w*_*i*_ = 0.12). The optimal model for the AUC of ACTH was the intercept only model (Δ_*i*_ = 0, *w*_*i*_ = 0.69), with only weak evidence for an effect of treatment (chronic stress group 13.4 [10.4; 16.5] pg.ml^-1^/min and control group 15.4 [12.3; 18.5] pg.ml^-1^/min, Δ_*i*_ = 1.59, *w*_*i*_ = 0.31).

#### Attention bias

The total duration of vigilance behaviour was lower and the frequency of transitions between vigilant and non-vigilant behaviour was higher in the chronic stress group (Table 2). These vigilance variables were best described by a model fitting the main effect of treatment, as opposed to a model fitting the intercept only (see Table 2 for AICc weights and model probabilities). The animals in the chronic stress group were twice as fast to start eating, and the latency to feed was best explained by a model fitting treatment as a factor (Table 2). Even though the chronic stress group consumed nearly twice as much food as the control group, the optimal model fitted the intercept only for food intake (Table 2). The number of zones crossed and vocalizations were also best explained by a model fitting the intercept only (Table 2).

**Table 2:** Predicted means, confidence intervals and model selection parameters for the behavioural variables in the attention bias test.

			Model Selection		
Variable	Treatment	Predicted means [CI]	Measures	Treatment	Intercept only
Duration vigilance	Chronic stress	164.1 [157.3; 171.0]	Δ*i*	0	3.5
behaviour (s)^$^	Control	176.1 [169.0; 183.2]	w*i*	0.85*	0.15
Mean bout frequency^$^	Chronic stress	7.0 [5.1: 8.9]	Δ*i*	0	2.35
	Control	4.0 [2.0; 6.0]	w*i*	0.76*	0.24
Looking at dog (s)^$^	Chronic stress	69.5 [56.4; 82.7]	Δ*i*	2.34	0
	Control	72.8 [59.6; 86.0]	w*i*	0.24	0.76*
Looking at food (s)^$^	Chronic stress	3.1 [1.1; 6.0]	Δ*i*	2.1	0
	Control	2.2 [0.6; 4.7]	w*i*	0.26	0.74*
Latency to eat (s)	Chronic stress	71.4 [40.0; 111.9]	Δ*i*	0	2.42
	Control	139.1 [91.9; 196.1]	w*i*	0.77*	0.23
Food intake (g)	Chronic stress	21.3 [6.5; 44.5]	Δ*i*	1.95	0
	Control	12.8 [2.6; 31.0]	w*i*	0.27	0.73*
Number of vocalizations	Chronic stress	1.8 [0.4; 4.3]	Δ*i*	1.09	0
	Control	0.6 [0.0; 2.2]	w*i*	0.37	0.63*
Number of zones crossed	Chronic stress	27.3 [16.8; 40.4]	Δ*i*	2.16	0
	Control	23.1 [13.5; 35.2]	w*i*	0.25	0.75*

^$^indicates variables analysed for the period without dog present, all other variables are for the entire period (with and without dog).

Δ_*i*_: differences in AICc values compared to the optimal model (having the lowest AICc value) within the set of models; *w*_*i*_: Akaike weight, the probability of the given model within the set of models.

*indicates the chosen optimal model

#### Pre-shearing judgement bias

**Fig 3:**
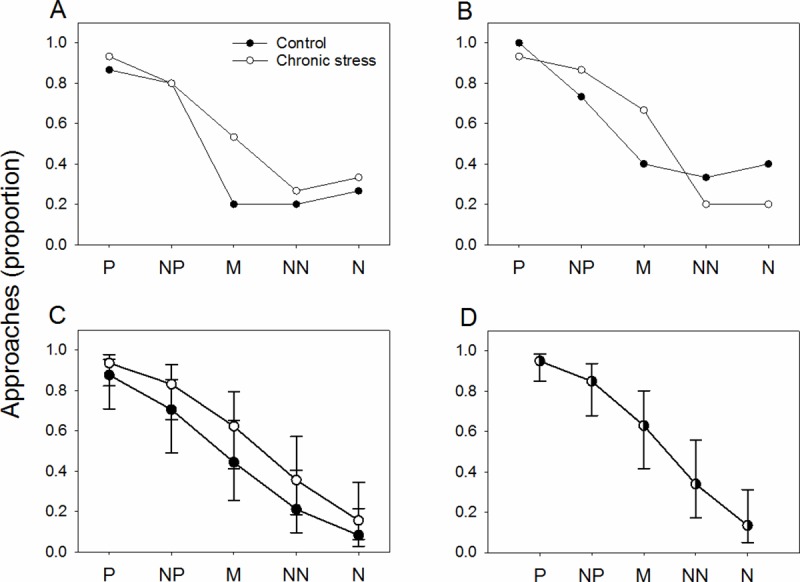
Judgement bias. (A) observed mean proportion of approaches to the five different locations during the judgement bias test after six days of chronic stress exposure (pre-shearing test) and (B) after nine days of chronic stress exposure and an acute shearing challenge (post-shearing test) and (C) mean proportion of approaches ± confidence interval from the GLMM model for the judgement bias test after six days of chronic stress exposure (pre-shearing test) and (D) after nine days of chronic stress exposure and an acute shearing challenge (post-shearing test) for the chronic stress and control group.

In the pre-shearing test, the majority of sheep approached the P locations, except for one sheep in the chronic stress treatment and two sheep in the control treatment (Fig 3A).

Spatial location/colour cue had the strongest effect on the likelihood that sheep would approach or not (model fitting a main effect of spatial location, Δ_*i*_ = 0, *w*_*i*_ = 0.52). However, the evidence that sheep from the chronic stress group were more likely to approach the locations was only slightly weaker (model fitting main effects of treatment and location, Δ_*i*_ = 0.78, *w*_*i*_ = 0.35, Fig 3A and 3C). The low Δ_*i*_ of the model fitting location and treatment suggest that this model can be considered almost equally likely as the model fitting location only. This is also demonstrated by the observation that the chronic stress group was nearly 3 times as likely to approach the middle ambiguous location.

### Effects of the acute shearing challenge

#### Plasma cortisol and ACTH responses to the shearing challenge

**Fig 4:**
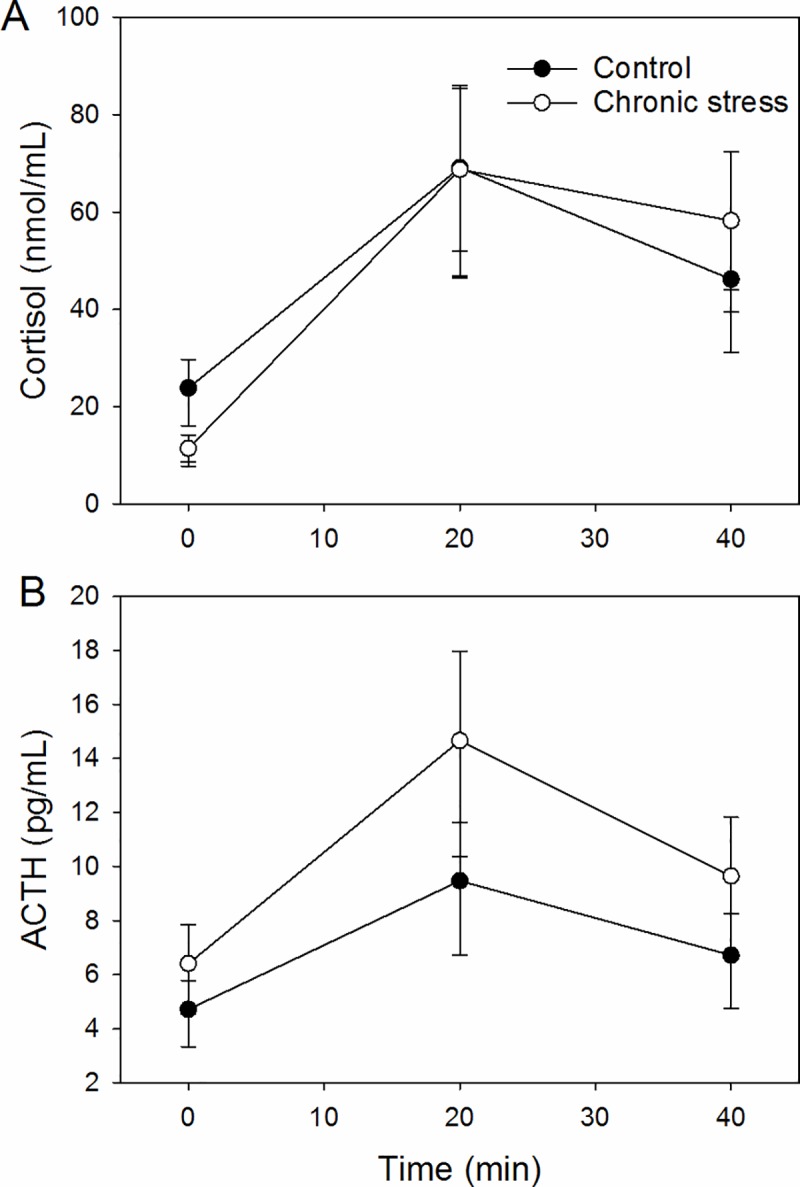
Effects of chronic stress treatment on predicted mean ± confidence interval plasma cortisol (A) and ACTH (B) concentrations following the acute shearing stressor.

Plasma cortisol concentrations in response to shearing over time were more affected in the chronic stress group (Fig 4A, model fitting a time x treatment interaction Δ_*i*_ = 0, *w*_*i*_ = 0.82, followed by a model fitting the main effect of time only, Δ_*i*_ = 3.24, *w*_*i*_ = 0.16). The optimal model for the AUC for cortisol fitted the intercept only (Δ_*i*_ = 0, *w*_*i*_ = 0.78), with only weak evidence for a main effect of treatment (chronic stress group 53.4 [41.7; 68.3] ng.ml^-1^/min and control group 53.2 [41.4; 68.1] ng.ml^-1^/min, Δ_*i*_ = 2.48, *w*_*i*_ = 0.23).

Plasma ACTH responses to shearing were higher in the chronic stress group (Fig 4B, optimal model fitting the main effects of time and treatment Δ_*i*_ = 0, *w*_*i*_ = 0.67, followed by a model fitting the main effect of time only Δ_*i*_ = 1.37, *w*_*i*_ = 0.33). The AUC for the ACTH responses was also higher in the chronic stress group (11.7 [9.2; 14.8] pg.ml^-1^/min) than in the control group (7.8 [6.2; 9.9] pg.ml^-1^/min, model fitting the main effect of treatment Δ_*i*_ = 0, *w*_*i*_ = 0.84, followed by the intercept only model Δ_*i*_ = 3.24, *w*_*i*_ = 0.17).

#### Body temperature responses to the shearing challenge

**Fig 5:**
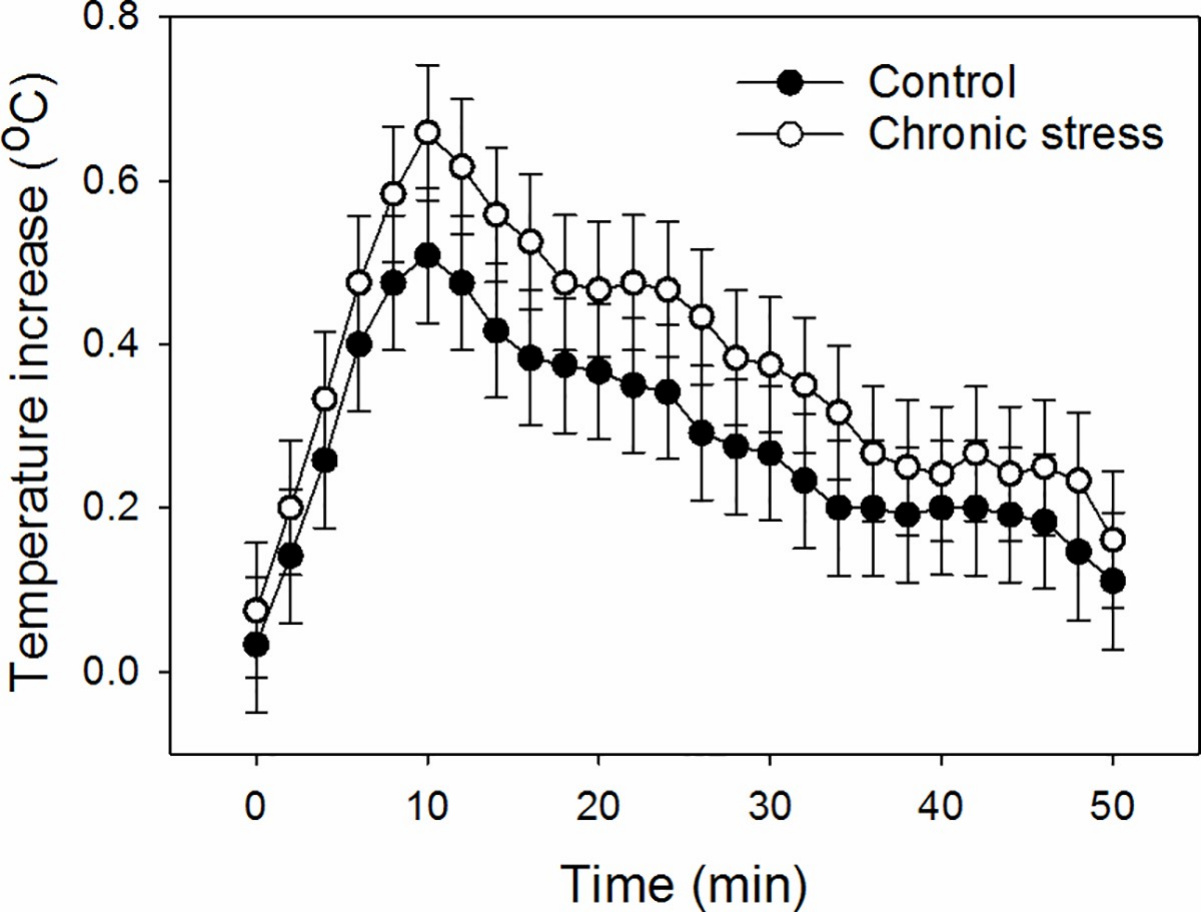
Effects of chronic stress treatment on predicted mean ± confidence interval internal body temperature following the acute shearing challenge.

The body temperature responses to the shearing challenge (Fig 5) were best explained by the time course of the experiment (optimal model fitted the main effect of time Δ_*i*_ = 0, *w*_*i*_ = 0.79) and we only found weak evidence for an effect of treatment (the second best model fitted the main effects of treatment and time Δ_*i*_ = 2.7, *w*_*i*_ = 0.21). The maximum increase in body temperature was higher in the chronic stress group (0.65 [0.59; 0.74]°C) than in the control group (0.53 [0.47; 0.60]°C, model with treatment as a main effect Δ_*i*_ = 0, *w*_*i*_ = 0.91, the second best model fitted the intercept only Δ_*i*_ = 4.57, *w*_*i*_ = 0.09).

#### Post-shearing judgement bias

In the post-shearing test, the majority of sheep approached the P locations, except for one sheep in the chronic stress treatment (Fig 3B).

Spatial location was also the main factor influencing the decision to approach a location or not (model fitting the main effect of location, Δ_*i*_ = 0, *w*_*i*_ = 0.60, Fig 3B and 3D). The evidence that sheep from chronic stress group were more likely to approach the location was weaker (model fitting main effects of location and treatment, Δ_*i*_ = 2.11, *w*_*i*_ = 0.21, Fig 3B and 3D)).

## Discussion

We aimed to induce chronic stress in sheep, characterized as a chronic disruption of the biological function [4, 5], by individual housing and rest deprivation. Sheep are a social species and separation from group members increases both behavioural and physiological responses indicative of stress [42, 43]. As expected, the chronic stress group spent more time lying down during comfort time than the control group, most likely to compensate for the restriction at other times. The disruption of lying behaviour would also have changed the daily resting pattern of the animals, because comfort time was only during daylight hours. We cannot be certain whether the lying disruption treatment also resulted in sleep deprivation, although there is a possibility that it did. It has been shown that abrupt changes in sleep patterns activate the HPA-axis [44]. The chronically stressed animals also showed signs of HPA-axis dysregulation as shown by both the CRH/AVP challenge and the acute shearing challenge. The chronic stress group lost slightly more body weight than the control group, which is a common sign of chronic stress in animals [45], but could also be explained by the slight variation in the diet between the control and chronic stress groups. Together the observed changes suggest that rest disruption and individual housing induced alterations in HPA-axis functioning that are indicative of a chronic stress state.

The HPA-axis is important in maintaining energy homeostasis. It is activated following many different types of stimuli, including several negative (i.e., social isolation and painful procedures) but also positive (i.e., food intake, sex) stimuli, which suggest that it mostly reflects physiological arousal and that HPA-axis activation in itself is not necessarily an indication of bad welfare [46]. However, chronic stress may lead to an increased allostatic load, due to the constant wear and tear on the physiological system [47]. This means that during long term stimulation of the HPA-axis by chronic stress, the HPA- axis is overloaded resulting in dysregulation. This can be further exacerbated by an acute challenge. An increased allostatic load has been shown to increase the risk of developing depressive or anxious emotional states [2, 6].

We found some signs of HPA-axis dysregulation in our chronic stress group: ACTH responses to the CRH/AVP challenge were reduced in the chronic stress group. However, ACTH responses were increased in the chronic stress group following the shearing challenge. It is possible that the ACTH response between the CRH/AVP and shearing challenges differed because the shearing challenge had to be psychologically perceived in order to activate the HPA-axis. The increase in ACTH responding to the shearing challenge, however, is a classic sign of down-regulation of the HPA-axis during chronic stress while maintaining responsivity to novel challenges [45, 48–50]. This down regulation of the HPA-axis may be protective against the detrimental effects of long-term high levels of circulating glucocorticoids in chronically stressed animals. The changes in cortisol concentrations in response to the CRH/AVP challenge and acute shearing challenge were less clear, and the chronic stress may have acted on the central regulation of the HPA-axis. For example, it is likely that central expression of CRH mRNA and AVP mRNA were increased in the chronic stress group [51]. The observed changes in HPA-axis functioning suggest an increased allostatic load in the chronic stress group.

The chronic stress group also showed reduced vigilance behaviour and was faster to approach the food, suggesting reduced attention towards a potential predator threat. General activity was unchanged in the attention bias test, so the quicker approach to the food is unlikely to be explained by a general increase in activity. We also found some evidence that the chronic stress group had a more optimistic judgement bias in the pre-shearing test compared to the control group, as shown by a slightly shifted curve while the steepness of the curve was not affected. This evidence, however, was relatively weak because our data could not fully differentiate between a model fitting the main effect of location only and a model fitting the main effects of treatment and location. The reduced vigilance behaviour and the seemingly more ‘optimistic’ judgement bias are contradictory to our hypothesis. Our results, however, are in agreement with other studies showing optimistic judgement biases following shearing and restraint challenges in sheep [52, 53], and following long-term mild stress in sheep [54] and horses [55]. One possible explanation for the optimistic judgement bias could be that the release from the negative environment itself led to the optimistic response [52]. Nevertheless, several other studies have found that long-term challenging and unpredictable housing induces a pessimistic judgement bias in sheep [56, 57] and rats [18] and an increased attention bias towards threat in anxious individuals is commonly found in human studies [26]. It is possible that the contradicting results are due to the type of challenge and differences in judgement bias assessment (e.g., spatial vs auditory task, types of stimuli used, or positive vs negative stimuli, or positive vs less positive stimuli etc.).

There are, however, alternative explanations for the reduced vigilance behaviour in the chronic stress group. In humans, it has been found that a challenge (competitive computer task) results in a rapid disengagement of attention towards negatively valenced words, but not neutral or positive words [58]. This attentional shift was associated with a stronger negative mood response to the challenge, suggesting that the attentional shift away from negative words was a response to the challenge-induced negative affect, and may be a way to cope with negative emotions [58]. Others have also reported shifts away from threatening information in non-anxious humans [59, 60]. In monkeys, attention was also shifted away from a threat (aggressive face) following an acute challenge [27]. These results suggest that attentional avoidance of threatening information may be a way to self-regulate the emotional state. However, an attention bias towards a predator threat (rather than attentional avoidance) would make more evolutionally sense in our context. Our sheep were very much used to dogs (daily presence of a dog while judgement bias training) and it is likely that sheep perceived the dog as only mildly aversive. Even though we did not assess attention towards or away from the threat directly in this study, the reduced vigilance behaviour could potentially be explained by the animals redirecting their attention towards the food reward and away from the threat, in an attempt to regulate their emotional state.

Alternatively, long-term challenges can lead to a general increase in motivational behaviours for rewarding stimuli, which in turn leads to increased consumption of highly palatable foods and drugs of abuse [61]. The consumption of “comfort foods” decreases cerebrospinal CRH concentrations and HPA-axis activity, suggesting that increased palatable food intake reduces the negative consequences of chronic challenges [62]. Individuals experiencing challenge may therefore increase palatable food intake in order to reduce their anxiety and make themselves feel better [63]. The ‘optimistic’ judgement bias in the chronic stress group in the pre-shearing test and the faster approach to the food in the attention bias test may be due to a general increase in motivation for a reward in an attempt to reduce the negative affective state.

We had also expected that sheep in the chronic stress group would have had a different judgement bias compared to the control group after shearing. There are different explanations possible for the lack of additional change in judgement bias in the chronic stress group following an acute challenge. It is possible that (1) animals either did not perceive the additional challenge as particular challenging, or that (2) our judgement bias test was not sufficiently sensitive to detect subtle differences in the affective state, that (3) their affective state had changed to the maximum and no further change was possible or that (4) animals had learned that the ambiguous locations were not reinforced in the post-shearing challenge [64]. The chronic stress group showed increased ACTH responses as well as a higher peak temperature following shearing compared to the control group. Stress-induced hyperthermia (SIH) is commonly observed when animals are exposed to challenges and may be indicative of the anxiogenic effects of the challenge [65]. Also, shearing is generally perceived as a major challenge in sheep [53, 66]. Therefore, the first explanation seems unlikely. The second explanation is possible, but would need further investigation. The third explanation is also a possibility and, if correct, suggests that the chronically stressed animals were unable to respond to the shearing challenge by altering their behaviour, which may be indicative of increased allostatic load. A high allostatic load results from the constant wear and tear on the physiological system due to repeated adaptation to challenges and it has been proposed that this can eventually result in physical and behavioural conditions such as mood disorders and cognitive dysfunction [1]. The welfare implications of this lack of adaptation to additional acute stressors in chronically challenged animals needs further investigation.

The housing and handling of the control group could also have impacted our results. For example, the animals in the control group were group housed and handled more positively in order to make the control animal’s environment as pleasant as possible. These differences in the human-animal relationship could potentially have affected the behaviour of the sheep [67–70]. On the other hand, the potential for multiple acute challenges (e.g. fitting temperature loggers, attention bias testing) may have influenced the control group responses to the judgement bias test. In an attempt to reduce some of these negative effects, we gave the control group a small food reward following a handling or experimental event that could potentially be perceived as challenging, because it has previously been shown that small positive events such as food rewards can alleviate some of the negativity experienced by animals [71, 72]. Animals also received one additional food reward a day while in the paddock; which may have led to a positive anticipatory response in the control group. Anticipatory behaviour to rewards is generally thought to be associated with a positive affective state [73, 74]. Despite all this, however, the control group animals responded in a similar manner in the judgement bias test following shearing as the chronic stress group after nine days of individual housing and rest deprivation. This could be due to a lack of sensitivity of the judgement bias test to stressor intensity, or to a ceiling effect of judgement bias that does not allow any further change after a certain “threshold” has been reached. Alternatively, the judgement bias test itself could have been experienced as mildly aversive compared to the normal pleasant housing, resulting in a more pessimistic judgement bias.

The negative reinforcer in our judgement bias test was a dog that was present throughout training and testing. We cannot exclude the possibility that the presence of olfactory cues during testing could have affected the responses of the sheep. The presence of reinforcers in test sessions has attracted discussion in the literature with no clear conclusion being reached as to whether reinforces should or should not be present during testing [75, 76]. Many judgement bias studies have been conducted where reinforcers are present during testing, for example a food bowl, either without food or with inaccessible food, placed at the ambiguous locations (for some examples see [31, 34, 71, 77–81]). Therefore, we cannot be entirely certain on exactly which cognitive processes were employed by the animals during the judgement bias test.

On the other hand, the perception of the dog’s olfactory cues itself may also have been influenced by the chronic stress treatment. It has been shown that the affective state can influence the perception of sensory information, called affective realism. For example, a negative mood results in increased perception of loudness [82] while unseen affectively positive stimuli increases the likelihood of a neutral face to be perceived as smiling [83]. We would like to stress that both the chronic stress and control groups in our study underwent identical training and testing regimens and were tested contemporaneously. With this balanced design we feel confident in our conclusion that differences between groups in the two test paradigms were a result of the challenging environment on affective state, whether or not the judgement bias test was confounded by olfactory cues.

There was also a high number of animals that did not reach the learning criteria for the judgement bias test (28 out of 60). We adapted the test by removing the bucket from the experiment, because it was too attractive for some animals, and this improved learning the no-go response. However, some other animals were scared of having to put their head through an open door to get their food reward, so this reduced the go responses in these animals. It is possible that we have selected animals with a certain temperament or personality (e.g., animals that are not afraid to collect a reward behind a door but are fearful enough not to risk getting confronted with a dog) and it has been shown that personality can affect performance in a judgement bias task in hens [84]. Furthermore, the order in which cues are presented in a judgement bias test also has an impact on the latency to approach the cues in hens [85]. Therefore, we chose to use two fixed order sequences for the cues in the judgement bias test, so that we could balance the two different orders across the treatments. There is a possibility that using this fixed order sequence of cues has influenced our results. We also cannot exclude the possibility of changed feeding motivation in the chronic stress group, which could potentially explain the faster approach to the food in the attention bias tests.

In conclusion, lying disruption combined with individual housing led to reduced plasma ACTH responses during the CRH/AVP challenge but increased ACTH responses following shearing. These changes in HPA-axis functioning are consistent with a chronic stress state. Furthermore, the chronic stress group was unable to mount a cortisol response to the shearing challenge reflective of the additional stress experienced. The chronic stress group also expressed a slightly more optimistic judgement bias compared to the control group (pre-shearing test) and approached the food quicker and showed reduced vigilance behaviour in the attention bias test. These cognitive biases are most likely due to an increased attention, expectation or motivation for rewarding stimuli induced by chronic stress. Despite nine days of exposure to challenges, judgement bias responses to the shearing challenge were similar in the chronic stress and the control groups. This lack of behavioural adaptation in the chronic stress group to the shearing challenge may be indicative of an increased allostatic load, and may increase the risk of compromised welfare in these animals.

## Supporting information

S1 File More details on judgement bias training. (PDF)

S2 File Judgement and attention bias data. (XLSX)

S3 File Cortisol, ACTH and temperature data. (XLSX)
