# Supplementary material for: Chronic stress influences attentional and judgement bias and the activity of the HPA axis in sheep
Source: PLoS One. 2019 Jan 30;14(1):e0211363. doi: 10.1371/journal.pone.0211363 (PMC6353200; doi:10.1371/journal.pone.0211363)
Supplement: S1 File — (PDF) [file pone.0211363.s001.pdf]

Judgement bias training

Sheep received a total of 46 training days, spread over a period of 2 months and 9 days. Training started by 4 initial habituation days in which food was providing in similar buckets at all five locations, and sheep were allowed to eat for 10 min in groups of three. On day 5, pairs of sheep were trained for 5 min with buckets placed at the two outer locations only. On day 6, sheep were pseudo-randomly assigned to a P location in either the outer left or outer right corner of the arena. The food reward was only located at the P location and sheep were individually trained to approach the P location two consecutive times. All other locations were inaccessible so that the sheep could only observe a reward bucket at the P location. A positive approach response was considered “correct” when the sheep stepped over the decision line at the entrance of the location with both front feet (Fig 1) and put its head inside the bucket (go response) within 30 s of entering the test arena. As soon as the animals consumed the reward, they were let out of the arena. If the animal did not make an approach within the maximum available time (30 s), they were let out of the arena (no-go response). Between day 7 and 10, the number of training runs was gradually increased to five consecutive runs per day to the P location, adapted from a previously used methodology [1]. From this point onwards, sheep received 5 training sessions per day. Sheep had to pass a performance criterion of 15 consecutive correct positive responses to the P location in order to proceed to negative training (days 10-14). Cue cards were not displayed above locations at this stage of training.

Negative training started on day 15. The N location was opposite the P location (e.g., if the P location was the outer left, then the N location was the outer right) and the bucket was placed at the N location, although it contained no food. A negative response was considered “correct” when the sheep did not approach the N location (no go response). If the sheep did not approach the N location it was let out of the arena after 30 s. An incorrect negative response (go response) was made when the sheep stepped over the decision line with both front feet (Fig 1), and the sliding door was lifted to reveal the dog. Sheep were let out of the arena after about 5 s of exposure to the dog, to prevent the sheep getting too stressed. Sheep were trained with the following order of P and N locations: PPNPP (day 15-20) and PNPNP (day 20-22), the ambiguous locations remained inaccessible. Once negative training had started, the dog always remained in position, even when sheep were being trained at the P location.

Many sheep continued to approach the N location, despite exposure to the dog when they approached the empty bucket. Therefore, we adapted the test by removing the bucket from view when sheep entered the test arena. The food reward at the P location was placed in a bowl hidden behind the sliding door. Sheep had to step over the decision line within 30 s of entering the arena in order for the door to be lifted and the food to be accessible. In order to facilitate discrimination between the different locations, we now attached the colour cue cards to the sliding doors at the different locations and the bucket was no longer used as a

cue. Sheep were pseudo-randomly assigned to either a 0% (white) or 95% brightness green cue at the P location. For each sheep, the cue at the N location was the opposite of the P location. Sheep received training at the P-location only between days 23 and 30.

Finally, sheep had to pass a training criterion in which they had to approach the P location 14 out of 15 times (go response), and not approach the N location at least 8 out of 10 times (no-go response) within 30 s. Sheep received 5 training sessions per day (between days 31-46) and therefore needed at least 5 different days to pass the criterion (total 25 sessions). The P and N cued locations were presented in the following order on the different days: PNPNP, PNNPP, NPNPP, PNNPP, NPPNP (repeated every 5 days), the ambiguous locations remained inaccessible.

Out of 60 sheep, 32 sheep passed all training criteria. Of these, two were randomly excluded and the remaining 30 sheep were used in the experiment. The reason for this was that based on previous experience, 15 animals per treatment is sufficient to observe statistically meaningful differences and it was unnecessary to subject additional animals to the experimental procedures.

1. Verbeek E, Ferguson D, Lee C. Are hungry sheep more pessimistic? The effects of food restriction on cognitive bias and the involvement of ghrelin in its regulation. Physiol Behav. 2014;123:67-75.
